# Sedentary Behavior Is Not Associated with Cardiometabolic Risk in Adults with Abdominal Obesity

**DOI:** 10.1371/journal.pone.0020503

**Published:** 2011-06-13

**Authors:** K. Ashlee McGuire, Robert Ross

**Affiliations:** 1 School of Kinesiology and Health Studies, Queen’s University, Kingston, Canada; 2 Department of Medicine, Division of Endocrinology and Metabolism, Queen's University, Kingston, Canada; Pennington Biomedical Research Center, United States of America

## Abstract

**Objective:**

The primary aim of this study was to determine whether time spent in sedentary behaviors (SED) was associated with 2-hour glucose and insulin resistance in adults with abdominal obesity. We also examined the association between light physical activity (LPA) and sporadic (accumulated in bouts <10 minutes in duration) moderate-to-vigorous physical activity (MVPA) with glucose metabolism.

**Methods:**

Participants were 135 inactive, abdominally obese adults recruited from Kingston, Canada. SED and physical activity were determined by accelerometry over 7 days and summarized as SED (accelerometer counts/min <100), LPA (counts/min 100–1951), and MVPA (counts/min ≥1952). A 75 g oral glucose tolerance test was used to ascertain 2-hour glucose; the homeostasis model of assessment was used to determine insulin resistance (HOMA-IR); lipid, lipoproteins and blood pressure were determined using standard protocols. Secondary analyses considered the association between SED and physical activity with other cardiometabolic risk factors.

**Results:**

Participants spent 627.2±82.9 min/d in SED, 289.0±91.7 min/d in LPA and 19.2±13.5 min/d in MVPA. Neither SED nor the physical activity variables were associated with 2-hour glucose or HOMA-IR (p>0.05). In secondary analyses, SED was not associated with any cardiometabolic risk factor (p>0.1); with the exception of blood pressure (p<0.05), LPA was not associated with any cardiometabolic risk factor (p>0.1); and MVPA was independently associated with total cholesterol and triglycerides (p<0.05).

**Conclusions:**

Objectively measured SED was not associated with 2-hr glucose or HOMA-IR. Our findings also suggest that the accumulation of LPA and sporadic MVPA is not associated with glucose metabolism in adults with abdominal obesity.

## Introduction

Sedentary behaviors (SED), which include activities such as television viewing or computer screen time, have gained widespread interest due to observations suggesting they have a negative impact on a variety of health outcomes [1]–[3]. Evidence from Healy *et al*. [4], [5] indicates that, independent of moderate-to-vigorous physical activity (MVPA), time spent in SED is positively associated with 2-hour glucose, waist circumference, and clustered metabolic risk in middle-aged adults. In contrast, Ekelund and colleagues [6] did not find a significant relationship between SED and insulin resistance in adults. Subsequently, others [7], [8] observe a univariate association of SED with select cardiometabolic risk factors however this association is not independent of MVPA or total activity. Although small differences in participant characteristics and accelerometry data reduction techniques exist between studies, there is no consistent pattern that would explain the disparate findings.

Whether time spent SED explains cardiometabolic risk beyond MVPA has important public health implications and thus we sought to clarify the relationships between SED, LPA, and MVPA with 2-hour glucose and insulin resistance in a population of inactive adults with abdominal obesity. Secondary analyses examined the association between SED, LPA, MVPA and other common cardiometabolic risk factors (triglycerides, total cholesterol, high-density lipoproteins, and blood pressure).

## Methods

### Participants

Participants for this study were men and women aged 35 to 65 years who were inactive, did not smoke, had an elevated waist circumference (defined as at least 102 cm in men and at least 88 cm in women), and a body mass index (BMI) between 25.0 to 39.9 kg/m^2^. Potential participants were excluded if they reported any physical impairment which would make physical activity difficult, or unsafe including history of myocardial infarction, stroke, coronary bypass surgery or angioplasty in the last 6 months; peripheral artery disease, unstable angina or ischemia; if they had diagnosed diabetes or were taking glucose-lowering medication; if they consumed >21 alcoholic drinks per week. The study was approved by the Queen’s University Health Sciences Research Ethics Board. All participants gave written informed consent before participation in the study.

### Anthropometric and Metabolic Tests

Body mass and height were measured to the nearest 0.1 cm and 0.1 kg, respectively, with participants dressed in standard T-shirts and shorts. These measures were used to calculate BMI (kg/m^2^). Waist circumference was obtained in a standing position using the mean of two measures acquired at the superior edge of the iliac crest measured to the nearest 0.1 cm.

Glucose tolerance was measured by means of a 2-hour glucose tolerance test the morning after an overnight fast. Blood samples were collected from the antecubital vein at the following time-points: 0, 30, 60, 90, and 120 minutes after ingestion of 75 g of Glucodex. Plasma glucose was determined using enzymatic methods on the Synchron LX® Systems (Bechkman Coulter, Inc., Brea, CA, USA). The homeostasis model assessment for insulin resistance (HOMA-IR) was used as a measure of insulin resistance and was calculated as fasting plasma glucose (mmol/L) x fasting serum insulin (µU/mL)/22.5. HOMA-IR was available for 99 participants. Blood samples to determine fasting triglycerides (TG), total cholesterol, and high-density lipoproteins (HDL) were also obtained in the morning after a 12- to 14-hour overnight fast. Serum total cholesterol, TG, and HDL levels were determined using standard enzymatic methods on the Synchron LX® Systems (Bechkman Coulter, Inc., Brea, CA, USA). Systolic blood pressure (SBP) and diastolic blood pressure (DBP) were measured using the BP Tru Blood Pressure Monitor (BPTru Medical Devices, Coquitlam, BC, Canada) in the morning after an overnight fast.

### Physical Activity by Accelerometry

Physical activity was measured with the Actigraph GT3X accelerometer (Actigraph, Pensacola, Florida, USA). Accelerometers were programmed to collect data in 1-minute epochs over 7 consecutive days and were worn on an elastic belt positioned over the right hip at all times except during water-based activities. Additionally, participants completed a log sheet indicating when they went to bed at night, woke up in the morning, and removed the accelerometer.

To be included in the analysis, participants were required to wear the accelerometer for at least four complete days (including one weekend day) within the monitoring period. A complete day was defined as at least 10 hours of wear time during the day. Wear time was calculated after extended periods of consecutive zero counts ≥60 minutes and sleep time (determined using both the participant logs and visual examination of the data) were excluded. Twelve participants did not meet the compliance criteria and were removed from the analysis. The accelerometer cutpoints in this study used to translate the ‘count’ value into an estimate of physical activity intensity were those developed by Freedson and colleagues [9]. LPA was defined as 100 to 1951 counts/min and MVPA as counts/min ≥1952. SED was defined as <100 counts/min, an arbitrary cutpoint commonly used [5], [7]. Activity accumulated during each complete day of monitoring was quantified in both absolute and relative terms: 1) average duration, in minutes per day, of each SED, LPA, MVPA, and total physical activity (LPA+MPVA; TPA), 2) percentage of time per day spent in each SED, LPA, MVPA, and TPA, 3) average minutes of MVPA per day accumulated sporadically (1 to 9 minutes), and 4) average minutes of MVPA per day accumulated in bouts (≥10 minutes). This duration is consistent with the consensus recommendation that physical activity be accumulated in intervals lasting 10 minutes or more for health benefit [10], [11]. Since periods of rest are common during activity (e.g., waiting for a light to change colour at a crosswalk during a walk), participants were required to spend at least 80% of the bout above the threshold value. For example, in a 10-minute bout of MVPA only 8 of those minutes would need to fall above 1952 counts/min. The bouted MVPA was used to determine whether participants were meeting physical activity guidelines.

### Statistical and Power Analyses

Descriptive characteristics are summarized as mean values ± standard deviations (SD). TG, HDL, HOMA-IR and all physical activity variables were logarithmically transformed due to skewed distributions. Associations between variables were examined using Pearson correlation coefficients. Differences between sex were determined using Independent Student’s T-tests. Sex differences in the relationship between physical activity and cardiometabolic risk factors were tested by adding the interaction terms to the regression models. With the exception of SBP and DBP, no differences were detected. Therefore analyses were collapsed across sex for all variables except blood pressure. To assess the association between SED, LPA, MVPA and cardiometabolic risk factors, linear regression models were used. Regression models using both the absolute and relative SED and physical activity variables were conducted. Only the covariates that significantly influenced the regression models were retained. Thus, when 2-hour glucose or HOMA-IR was the outcome variable the following models were used: 1) adjusted for time accelerometer worn and 2) adjusted for time accelerometer worn and waist circumference. To assess the association between SED, LPA, MVPA and other common cardiometabolic risk factors (total cholesterol, TG, HDL, SBP and DBP), the following models were used: 1) adjusted for time accelerometer worn, 2) adjusted for time accelerometer worn, sex, age, waist circumference, and 3) adjusted for time accelerometer worn, sex, age, waist circumference, and the other physical activity variables. In all regression models, multicollinearity was controlled for using the variance inflation factor and tolerance statistic. Significance was set at p<0.05 for main effects and at p<0.1 for interaction. All statistical analyses were performed using SPSS 18.0 software (SPSS, Chicago, IL).

Power calculations were based on our primary outcomes (2-hour glucose and HOMA-IR). In our convenience sample of 135 with 2-hour glucose values, we estimate that we have 95% power to detect a correlation of 0.3 with an alpha of p<0.05. In our sample of 99 participants with HOMA-IR data we have 85% power to detect a correlation of 0.3 at an alpha of p<0.05.

## Results

The participant characteristics are shown in Table 1. Men had a higher waist circumference, TG, and DBP (p<0.01) whereas women had higher HDL (p<0.001). Men participated in more MVPA than women (p<0.01).

**Table 1:** Participant characteristics.

Variable	Men (n = 43)	Women (n = 92)	Total (n = 135)
Age (years)	54.3±8.3	52.5±7.3	53.1±7.6
Body mass index (kg/m^2^)	32.9±3.4	32.9±5.0	32.9±4.6
Waist Circumference (cm)	115.9±8.4	107.3±11.3*	110.0±11.2
2-hour Glucose (mmol/L)	7.0±2.0	6.9±1.7	6.9±1.8
HOMA-I 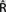	2.7±1.6	2.2±1.7	2.3±1.7
Total Cholesterol (mmol/L)	5.0±0.9	5.3±1.1	5.2±1.0
Triglycerides (mmol/L)	1.8±1.0	1.4±0.7**	1.5±0.8
High-Density Lipoproteins (mmol/L)	1.0±0.3	1.3±0.3*	1.2±0.4
Systolic Blood Pressure (mmHg)	123±12	121±15	122±14
Diastolic Blood Pressure (mmHg)	81±7	78±9**	79±8
**Physical Activity (Accelerometry)**			
Wear Time (min/d)	970.9±127.9	940.2±88.1	950.0±103.0
Average Daily Intensity (counts/min)	536.1±199.7	535.8±143.6	535.9±162.8
Sedentary Behavior (min/d)	647.8±72.0	617.8±86.2	627.2±82.9
Average Sedentary Intensity (counts/min)	10.7±3.0	11.3±2.7	11.0±2.8
Time in Sedentary Behavior (%)	67.6±8.7	65.8±7.8	66.4±8.1
Light Physical Activity (min/d)	282.6±117.5	292.0±77.4	289.0±91.7
Average Light Activity Intensity (counts/min)	556.4±78.9	531.7±73.9	539.6±76.1
Time in Light Activity (%)	28.6±7.8	31.0±7.2	30.2±7.5
Moderate-to-Vigorous Physical Activity (min/d)	23.9±17.4	17.0±10.6**	19.2±13.5
Average Moderate-to-Vigorous ActivityIntensity (counts/min)	2886.7±353.5	2765.5±374.1	2804.1±370.7
Time in Moderate-to-Vigorous Activity (%)	2.4±1.5	1.8±1.1	2.0±1.3
Total Physical Activity (min/d)	306.5±129.6	309.0±81.4	308.2±98.8

Data are means ± SD. Significant difference between sex: *(p<0.001), **(p<0.01) 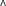
n = 99 for HOMA-IR; homeostasis model assessment for insulin resistance.

Accelerometers were worn for a median of 7 days and for 16 hours per day on average. Participants spent 66% of their waking hours in SED, 30% in LPA, and only 2% in MVPA. Although all participants were confirmed as inactive according to the consensus recommendation that adults accumulate 30 minutes of daily MVPA in bouts of ≥10 minutes, approximately 5 hours of LPA and 20 minutes of sporadic MVPA were accumulated daily (Table 1).

When expressed as a percentage of wear time, SED was negatively correlated with LPA (r = −0.85, p<0.01) and MVPA (r = −0.39, p<0.05) whereas LPA and MVPA were positively correlated (r = 0.35, p<0.01). SED, LPA, and MVPA were not associated with waist circumference (p>0.1). Waist circumference was positively correlated with 2-hour glucose (r = 0.18, p<0.05) and HOMA-IR (r = 0.52, p<0.01).

**Figure 1:**
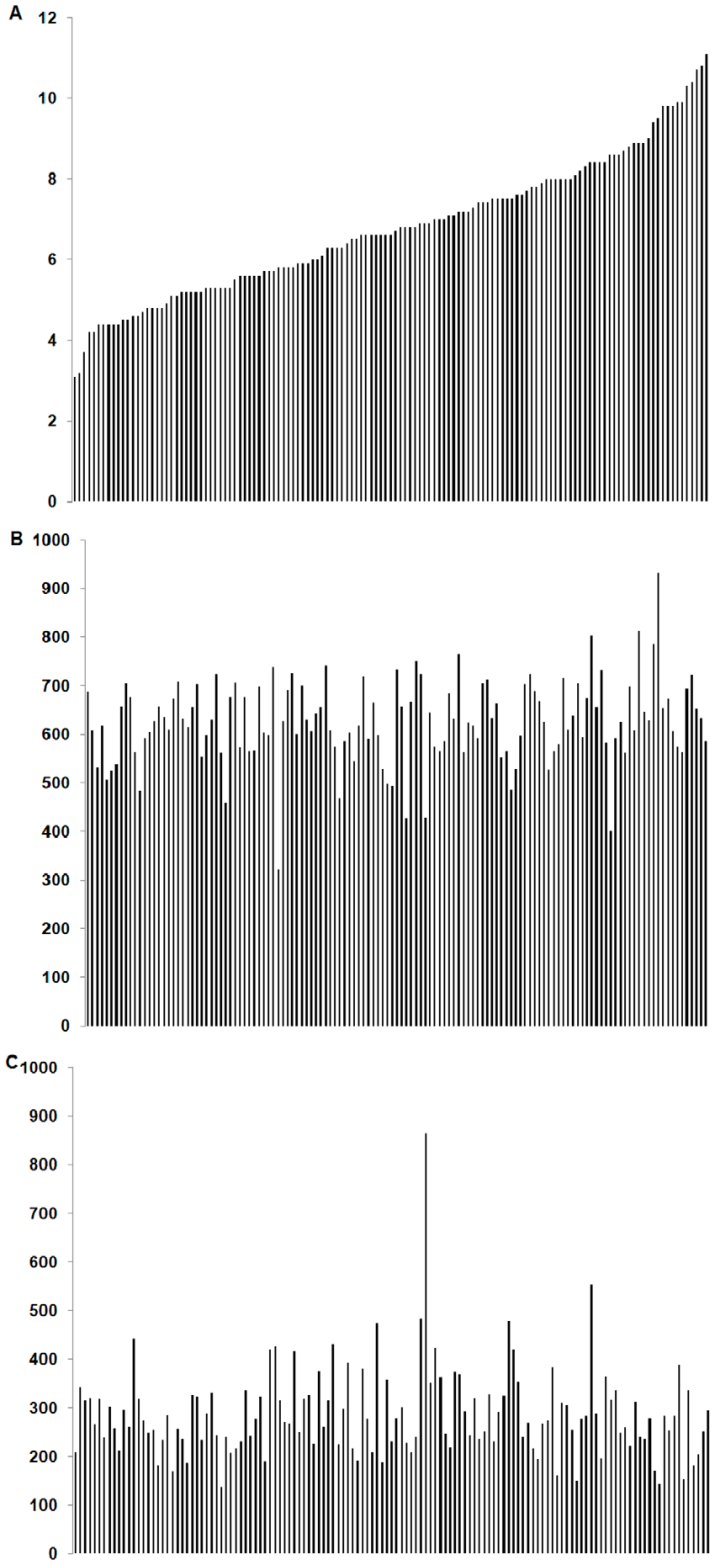
Associations of 2-hour glucose, sedentary behavior and physical activity. Association of (A) 2-hour glucose (mmol/L) with (B) sedentary behavior (min/d) and (C) light physical activity (min/d) for each individual participant.

Neither SED nor the physical activity variables were associated with 2-hour glucose or HOMA-IR (p>0.05). Results were the same regardless of whether SED and the physical activity variables were expressed in absolute or relative terms (Tables 2 and 3). Results were not different for TPA (data not shown). To further illustrate the relationship between SED, PA and 2-hour glucose, individual data were plotted in ascending order for 2-hour glucose along with the corresponding data, in the same order, for both SED and LPA. As illustrated in Figure 1, with increasing 2-hour glucose, there is no clear corresponding increase in SED or decrease in LPA.

**Table 2:** Regression analyses of sedentary behavior and physical activity (expressed as minutes per day) with 2-hour glucose and HOMA-IR.

2-Hour Glucose				HOMA-IR		
(N = 135)				(N = 99)		
	B (95% CI)	P	R^2^	B (95% CI)	P	R^2^
**Model 1**						
SED (min/d)	0.00 (−0.00 to 0.01)	0.31	0.00	0.00 (−0.00 to 0.00)	0.46	0.00
LPA (min/d)	−0.14 (−0.28 to 0.00)	0.06	0.03	−0.01 (−0.04 to 0.03)	0.74	0.00
MVPA (min/d)	−0.13 (−0.35 to 0.09)	0.23	0.01	−0.00 (−0.05 to 0.05)	0.94	0.00
**Model 2**						
SED (min/d)	0.00 (−0.00 to 0.01)	0.39	0.02	0.00 (−0.00 to 0.00)	0.71	0.29
LPA (min/d)	−0.12 (−0.26 to 0.02)	0.10	0.04	−0.01 (−0.05 to 0.02)	0.39	0.29
MVPA (min/d)	−0.14 (−0.35 to 0.08)	0.22	0.03	−0.01 (−0.05 to 0.04)	0.80	0.29

Model 1: adjusted for time accelerometer worn.

Model 2: adjusted for time accelerometer worn and waist circumference.

SED; sedentary behavior, LPA; light physical activity, MVPA; moderate-to-vigorous physical activity.

**Table 3:** Regression analyses of sedentary behavior and physical activity (expressed as percentage wear time) with 2-hour glucose and HOMA-IR.

2-Hour Glucose				HOMA-IR		
(N = 135)				(N = 99)		
	B (95% CI)	P	R^2^	B (95% CI)	P	R^2^
**Model 1**						
SED (%)	0.01 (−0.03 to 0.05)	0.54	0.01	0.00 (−0.00 to 0.01)	0.52	0.00
LPA (%)	−0.02 (−0.06 to 0.02)	0.38	0.00	0.00 (−0.01 to 0.01)	0.94	0.00
MVPA (%)	−0.35 (−0.99 to 0.29)	0.28	0.00	−0.00 (−0.11 to 0.11)	0.96	0.00
**Model 2**						
SED (%)	0.01 (−0.03 to 0.05)	0.63	0.02	0.00 (−0.00 to 0.01)	0.39	0.30
LPA (%)	−0.02 (−0.06 to 0.02)	0.43	0.02	−0.00 (−0.01 to 0.01)	0.65	0.29
MVPA (%)	−0.34 (−0.97 to 0.29)	0.29	0.03	−0.01 (−0.11 to 0.08)	0.78	0.29

Model 1: adjusted for time accelerometer worn.

Model 2: adjusted for time accelerometer worn and waist circumference.

SED; sedentary behavior, LPA; light physical activity, MVPA; moderate-to-vigorous physical activity.

In secondary analyses, SED was not associated with any of the cardiometabolic risk factors (p>0.1). LPA was not significantly associated with total cholesterol, TG, or HDL (p>0.1) but was a significant independent predictor of both SBP and DBP (p<0.01) in men, explaining 16% and 22% of the variance, respectively and DBP in women (p<0.05). MVPA was significantly associated with total cholesterol and TG after control for time accelerometer worn, age, sex, waist circumference, and other physical activity variables (p<0.05) however the variance explained by MVPA was less than 10%. Results with TPA as the independent variable were the same as those for LPA (data not shown). Similarly to the primary analyses, results were the same when SED and the physical activity variables were expressed as either absolute or relative values with the following exception: when LPA and TPA were expressed as a percentage of wear time neither were associated with either SBP or DBP in either sex (p>0.1).

## Discussion

The primary finding of this study was that time spent in SED was not associated with glucose metabolism in inactive men and women with abdominal obesity. These results combined with a prior investigation [6] question the unique contribution of SED to cardiometabolic risk in adults. We also observed that the average accumulation of approximately 5 hours of LPA plus an additional 20 minutes of sporadic MVPA on a daily basis was not associated with 2-hour glucose or HOMA-IR in this cross-sectional analysis. This observation supports the consensus recommendation that for health benefit physical activity should be accumulated in bouts of at least 10 minutes.

Our primary finding is consistent with observations by Ekelund *et al*. [6] who report that SED is not a significant predictor of HOMA-IR in overweight men and women with a family history of type 2 diabetes. However, our results counter those of Healy and colleagues [4] which suggest that, independent of both LPA and MVPA, SED is negatively associated with 2-hour glucose in men and women recruited from the general Australian population (AusDiab Study). Somewhat consistent with Healy *et al*. [4], data from the RISC Study [8] and ProActive Trial [7] indicate a significant relationship between SED and HOMA-IR in univariate analyses, however after statistical control for total activity or MVPA these associations are no longer significant. Thus at present the relationship of SED with glucose metabolism in adults is unclear.

The participants in our study had a more deleterious cardiometabolic risk factor profile and were more obese than participants in the prior studies however this is not likely to affect the relationship between SED, physical activity, and cardiometabolic risk factors. Further, there is no distinct difference in participant characteristics or statistical analyses between the investigations that may explain the differences in findings. For example, the participants in our study are similar in age to those in the AusDiab study [4], [5] whereas the cardiometabolic risk factor profile is very similar between participants in the ProActive Trial [6] and AusDiab study [4], [5].

Alternatively, the discrepancies may be explained in part by measurement limitations and inconsistent PA profiling.

Although the use of accelerometry to objectively measure SED and physical activity represents a major advance in SED and physical activity research, these devices are not without limitations. For example, accelerometers are unable to detect variations in SED due to fidgeting or provide context to the behavior and cannot capture certain activities such as cycling. Therefore differences between populations in SED pursuits and activities not captured by the accelerometer may influence relationships of SED and physical activity with health outcomes. Additionally, there are currently no published studies comparing the output of earlier Actigraph models to the GT3X utilized in the present investigation. It is possible that there is a difference between models in the ability to detect activity at the lower end of the movement spectrum. Consequently, the amount of time spent in SED and LPA may be influenced.

In the literature there is also considerable inconsistency in the presentation of physical activity patterning despite the use of common cutpoints to classify SED and physical activity. For example, it is unclear whether the participants in the AusDiab Study accumulated MVPA sporadically or in bouts. Given the consensus recommendation that MVPA be accumulated in bouts of at least 10 minutes to confer benefit across a wide range of health outcomes [10], [11], the associations between MVPA and cardiometabolic risk factors could differ depending on how the participants accrued daily MVPA and could explain why others find that MVPA is a significant predictor of both 2-hour glucose [4] and HOMA-IR [6]. Thus, it would be helpful to the field of SED and physical activity research to standardize how variables are reported as this would enable direct comparisons between studies and help to eliminate confusion.

In this study secondary analyses revealed that SED was not associated with other common cardiometabolic risk factors in abdominally obese, inactive adults. Although Ekelund and colleagues [7] report similar findings, these results do not support recent evidence derived from animal models where, after only one day of hind limb suspension (to remove ambulation), a significant decrease in lipoprotein lipase activity, TG uptake, and HDL concentrations is noted [12]. We also observed that with few exceptions, LPA and MVPA were not associated with other common cardiometabolic risk factors. Whereas our results are similar to some [5], [8], they counter others [5], [7]. Substantial differences in populations studied, methodology and statistical analyses that may influence the results were not noted. Thus, it is difficult to reconcile the inconsistent results.

The findings from our study were derived from a relatively homogenous sample of middle-aged, inactive adults with abdominal obesity. However, in North America approximately 34% of adults are obese [13] and at least 90% do not meet consensus recommendations for physical activity [14]. Thus, our results are generalizable to a large percentage of the adult population.

In summary, whether time spent sedentary independently predicts cardiometabolic risk in adults remains to be resolved. Given the public health implications, it is important that this question be answered and thus, it would be helpful if future investigations using accelerometry employed standardized methodology in order to facilitate direct comparison between studies.
